# Relationship between contrast-induced nephropathy and long-term mortality after percutaneous coronary intervention in patients with chronic coronary total occlusion

**DOI:** 10.1590/1806-9282.20220283

**Published:** 2022-08-19

**Authors:** Tuncay Güzel, Adem Aktan, Muhammed Demir, Mehmet Özbek, Burhan Aslan

**Affiliations:** 1 Diyarbakir Gazi Yaşargil Training and Research Hospital, Department of Cardiology – Diyarbakır, Turkey.; 2 Mardin Training and Research Hospital, Department of Cardiology – Mardin, Turkey.; 3 Dicle University Faculty of Medicine, Department of Cardiology – Diyarbakır, Turkey.

**Keywords:** Coronary occlusion, Acute renal injury, Mortality, Atherosclerosis

## Abstract

**OBJECTIVE:**

Intervention in chronic total occlusion lesions involves long procedure time, a serious contrast load, and complex procedures. In this study, we aimed to investigate mortality rate of patients who had procedural coronary angiography done for chronic total occlusion lesions in coronary angiography series and who developed contrast-induced nephropathy.

**METHODS:**

A total of 218 patients with chronic total occlusion lesion in at least one coronary artery, from three different medical centers, who underwent procedural coronary angiography were recruited for the study. Patient population was divided into two groups: those who developed contrast-induced nephropathy and those who did not. Mortality due to all causes was investigated between both groups throughout a 100-month follow-up.

**RESULTS:**

Mean age of patients with incidence of contrast-induced nephropathy was 66.7±11.8, and 23.8% of them were comprised by female. We found a significantly higher mortality in long-term follow-up in the patient group with contrast-induced nephropathy (42.9 vs. 57.1%, p=<0.001). According to Kaplan-Meier analysis performed additionally, survival during follow-up was significantly shorter in this group and, in logistic regression analysis, it was an independent predictor of mortality (OR 11.78; 95%CI 3.38–40.9).

**CONCLUSION:**

We identified that the development of contrast-induced nephropathy is associated with long-term mortality. It might be possible to reduce adverse events with prophylactic approaches before the procedure and close follow-up of such patients after the procedure.

## INTRODUCTION

Atherosclerosis forms the basis of coronary artery disease, and it is a progressive process accompanied by inflammation, showing systemic involvement^1^. Chronic total occlusion (CTO) of coronary arteries is described as complete occlusion of vein lumen for a minimum of 3 months (TIMI 0 flow)^2^. CTO is commonly seen in the range of 18–52% of patients undergoing coronary angiography (CAG)^3,4,5^. Revascularization of CTO lesions by percutaneous coronary intervention (PCI) leads to improved left ventricular functions and positive contributions to mortality. Many studies have demonstrated clinical benefits of revascularization of CTO lesions^6,7,8^. Recently, progress has been made in PCI treatment methods in CTO lesions^9,10^. With increased PCI experience in CTO lesions, antegrade and retrograde methods have increased the success rate of procedures^10^. Nevertheless, success rates have decreased due to complex CTO lesions, long-term exposure to radiation, and the use of large volumes of contrast agent^11^. Contrast-induced nephropathy (CIN) is described as an increase of 0.5 mg/dL or ≥25% in baseline serum creatinine levels after 48–72 h of exposure to contrast agent. Its incidence is expected to be in the range of 1–6% in general population and it is even higher relative to protein kinase G (PKG)^12^. However, frequency of CIN has increased in patients with impaired kidney function, and this percentage was more than 50% in high-risk patients^13^.

Patients with CTO who undergo revascularization are usually older and more likely to have diabetes, multiple coronary artery disease, low left ventricle ejection fraction (LVEF), and poor renal function^14^. Accordingly, such patients are at higher risk for CIN, one of the major complications after the procedure^15^.

Previous studies reported an incidence of CIN after PCI of about 6–7% in CTO lesions^11^. CIN accounts for about 11% of acute renal failure (ARF) cases and is the third leading cause of hospital-acquired ARF. Hospital mortality rate has been reported to be 22% of the patients developing CIN and 1.4% of those who does not^16^.

Studies in the literature mainly focus on scoring systems estimating development of contrast nephropathy and predictors; in contrast, in this study, we aimed to investigate long-term mortality rate in patients with CTO lesions who had procedural CAG and developed CIN.

## METHODS

A total of 218 patients with CTO lesion in at least one coronary artery, from three different medical centers, who underwent procedural CAG between February 2010 and April 2012 were recruited for this study. It was planned as a multicenter, retrospective, and cross-sectional study. CAG images of the patients were examined and chosen by three different cardiologists. Demographic characteristics, laboratory findings, echocardiography findings, and follow-up data of the patients were obtained from the hospital’s database. Mortality throughout 100-month follow-up process was considered primary end point. Study consent was obtained from local ethics committee in accordance with 2013 Declaration of Helsinki. Inclusion criteria were patients who had CTO in at least one coronary artery and underwent procedural CAG, EF≥40, and age between 18 and 90 years. Exclusion criteria were EF≤40, end-stage kidney failure, history of renal transplantation, pregnancy, hypotension, the use of intra-aortic balloon pump, rheumatic and connective tissue diseases, malignancy, and active infection. A non-ionic, low-osmolality contrast agent was used in all patients included in the study. Angioplasty technique and the amount of contrast agent used were at the discretion of the physician. All patients included in the study were given hydration with a liquid containing 1200 mg of *N*-acetyl cysteine in a minimum 1000 cm^3^ of 0.9% isotonic sodium chloride solution before and after the procedure.

### Definitions

Blood samples were collected from anterior facet of anterior arm of all study patients while they were in supine position after their admission to cardiology clinic. Blood parameters were measured in the serum separated by centrifuging at 3000×g cycles at room temperature. Patients with blood pressure above 140/90 were considered hypertensive, and those below 90/60 were considered hypotensive. Patients with low-density lipoprotein (LDL) value of 160 mg/dL were considered to have hyperlipidemia. In echocardiography laboratory, patients’ LVEF were measured using transthoracic 2D echocardiography (Vivid S6, GE Medical Systems, USA). CIN was described as an increase of 0.5 mg/dL or ≥25% in serum creatinine concentration in the first 48–72 h after CAG. A glomerular filtration rate (eGFR) of <60 mL/min/1.73 m^2^, determined by Cockcroft-Gault formula, was considered renal failure.

### Statistical analysis

The IBM SPSS version 24.0 software package was utilized for analysis. Baseline continuous variables were presented as means (standard deviation [SD]) or median with the first and third quartiles (Q1–Q3) depending on the distribution of data. Categorical variables were described as frequency and percentage. Normal distribution of variables was analyzed through Kolmogorov-Smirnov and Shapiro-Wilk tests. Logarithmic transformation was made since the variables showed abnormal wide distributions for blood parameters, namely, platelets, glucose, neutrophils, and lymphocytes. Kaplan-Meier test was used to analyze the correlation between development of CIN and survival during a 100-month follow-up period. Continuous variables were compared through Student’s t-test or Mann-Whitney U test, as appropriate. Univariable analysis was applied for continuous variables, while chi-square or Fisher’s exact test for categorical variables. Both univariable and multivariable logistic regression analyses were performed to evaluate the parameters affecting the development of CIN. Univariable and multivariable Cox regression analyses were used to identify predictors of mortality associated with all reasons. Only parameters with p≤0.1 were included in the evaluation during multivariate regression analyses. A p<0.05 was considered statistically significant for all tests.

## RESULTS

Demographic data and accompanying diseases of all the study patients are summarized in Table 1. Mean age of patients who developed CIN was significantly higher than those who did not (66.7±11.8 vs. 62.4±11.1, p=0.027). Development of CIN was significantly higher in peripheral artery patients and in those with low EF (14.3 vs. 3.4%, p=0.005 and 43.7±11.8 vs. 49±10.6, p=0.003) (Table 1).

**Table 1.** Demographic, clinical characteristics, and laboratory parameters of groups.

Parameters	Patients developing CIN (n=42)	Patients not developing CIN (n=176)	p-value
Age, years	66.7±11.8	62.4±11.1	**0.027**
Female, n (%)	10 (23.8)	49 (27.8)	0.597
Hypertension, n (%)	19 (45.2)	59 (33.5)	0.155
Hyperlipidemia, n (%)	3 (7.1)	8 (4.5)	0.490
Diabetes mellitus, n (%)	17 (40.5)	52 (29.5)	0.171
Chronic kidney disease, n (%)	4 (9.5)	6 (3.4)	0.089
Smoking, n (%)	15 (35.7)	43 (24.4)	0.137
Cerebrovascular disease, n (%)	3 (7.1)	3 (1.7)	0.053
Peripheral arterial disease, n (%)	6 (14.3)	6 (3.4)	**0.005**
Left ventricle ejection fraction, n (%)	43.7±11.8	49±10.6	**0.003**
Mortality, n (%)	24 (57.1)	31 (42.9)	**<0.001**
Follow-up period (IQR)	42 (21.2-65)	33 (21-57.7)	0.539

CIN: contrast-induced nephropathy; IQR: interquartile range. Bold indicates significant value.

We found a significantly higher mortality rate in the CIN patient group (57.1 vs. 42.9%, p=<0.001) (Table 1).

The patients are compared for laboratory data in Table 2. C-reactive protein (CRP) and LDL values were significantly higher in the CIN group [0.62 (0.30–6.8) vs. 0.38 (0.10–2.5), p=0.029 and 117±33.8 vs. 103±38, p=0.035] (Table 2).

**Table 2.** Logistic regression analysis of development of contrast-induced nephropathy in 100-month follow-up.

Parameters	Patients developing CIN (n=42)	Patients not developing CIN (n=176)	p-value
White blood cell, 10^3^/μL	10.0±3.5	9.6±3.5	0.540
Hemoglobin, gr/dL	13.4±1.9	13.6±1.8	0.462
Neutrophile, 10^9^/L	7.1±3.4	6.6±3.5	0.409
Lymphocyte, 10^9^/L	1.9±0.86	2.1±0.89	0.087
Platelet, 10^3^/μL	251±91	245±70	0.641
Glucose, mg/dL	134 (100-254)	118 (99-187)	0.389
Creatinine, mg/dL	1.09±0.32	1.00±0.46	0.202
Glomerular filtration rate, mL/min	74.8±25.0	82.9±24.5	0.058
Sodium, mEq/L	136.4±3.5	135.5±10.5	0.576
Potassium, mEq/L	4.3±0.65	4.6±2.6	0.459
Albumin, mg/dL	3.5 (3-3.9)	3.7 (3.3-3.9)	0.182
C-reactive protein, mg/dL	0.62 (0.30-6.8)	0.38 (0.10-2.5)	**0.029**
Total cholesterol, mg/dL	188±41	179±50	0.281
HDL, mg/dL	37.5±7.4	40±11	0.151
LDL, mg/dL	117±33.8	103±38	**0.035**
Triglyceride, mg/dL	137 (96-198)	145 (98-213)	0.851

CIN: contrast-induced nephropathy; HDL: high-density lipoprotein; LDL: low-density lipoprotein. Bold indicates significant value.

Regression analyses of CIN development during a 100-month follow-up period by demographic characteristics, clinical properties, and laboratory parameters are summarized in Table 3. Multivariable analysis was applied to parameters with a p≤0.1 after univariable analysis (Table 3).

**Table 3.** Regression analyses of development of CIN during a 100-month follow-up period by demographic characteristics, clinical properties, and laboratory parameters.

Parameters	Univariable analysis		Multivariable analysis	
	OR (95%CI)	p-value	OR (95%CI)	p-value
Age	1.03 (1.00–1.06)	**0.029**	1.04 (1.0–1.08)	**0.048**
Gender	0.81 (0.37–1.77)	0.598	–	
Hypertension	0.61 (0.30–1.20)	0.157	–	
Diabetes mellitus	0.61 (0.30–1.23)	0,173	–	
Chronic kidney disease	0.33 (0.09–1.24)	0.103	0.37 (0.06–2.15)	0.274
Smoking	0.58 (0.28–1.19)	0.140	–	
Peripheral arterial disease	4.72 (1.44–15.48)	**0.010**	0.13 (0.02–0.75)	**0.023**
Left ventricle ejection fraction	0.95 (0.92–0.98)	**0.004**	0.96 (0.92–1.0)	0.054
Hemoglobin, gr/dL	0.93 (0.77–1.12)	0.461	–	
Platelet, 10^3^/μL	1.30 (0.09–17.3)	0.839	–	
Glucose, mg/dL	3.65 (0.74–17.8)	0.109	1.64 (0.21–12.4)	0.629
C-reactive protein	1.13 (1.03–1.25)	**0.007**	1.10 (1.0–1.22)	**0.048**
Total cholesterol	1.0 (0.99–1.01)	0.281	–	
Number of sick vessels (multiple total vessels are present/not present)	0.49 (0.21–1.12)	0.094	0.53 (0.18–1.52)	0.240
Rentrop (good/poor)	1.31 (0.66–2.60)	0.436	–	

Bold indicates significant value.

Cox regression analysis performed among all factors affecting mortality in CTO patient population revealed that age, diseases such as hypertension (HT), diabetes mellitus (DM), and CIN, EF and hemoglobin results were significant in univariable analysis. In multivariable analysis (parameters with p≤0.1 were included), it was concluded that CIN incidence in CTO patient population is an important and independent predictor of mortality among all parameters (OR 3.02; 95%CI 1.41–6.45; p=0.004) (Table 4).

**Table 4.** Cox proportional hazard regression analysis of risk of death regression model during 100-month follow-up in the study population.

Parameters	Univariable analysis		Multivariable analysis	
	OR (95%CI)	p-value	OR (95%CI)	p-value
Age	1.04 (1.01–1.06)	**0.001**	1.00 (0.97–1.04)	0.629
Gender	0.89 (0.50–1.58)	0.707	–	
Hypertension	1.71 (1.01–2.91)	**0.046**	1.59 (0.78–3.23)	0.197
Diabetes mellitus	1.83 (1.07–3.11)	**0.025**	1.18 (0.40–3.48)	0.753
Chronic kidney disease	2.26 (0.90–5.69)	0.083	0.80 (0.15–4.12)	0.792
Smoking	1.04 (0.59–1.85)	0.876	–	
Peripheral arterial disease	1.88 (0.80–4.40)	0.145	–	
Left ventricle ejection fraction	0.95 (0.93–0.97)	**<0.001**	0.99 (0.95–1.02)	0.545
CIN development	3.14 (1.83–5.37)	**<0.001**	3.02 (1.41–6.45)	**0.004**
Hemoglobin	0.81 (0.70–0.93)	**0.003**	0.80 (0.65–0.98)	**0.031**
Neutrophile	2.80 (0.82–9.55)	0.100	–	
Lymphocyte	0.16 (0.04–0.65)	**0.010**	0.35 (0.05–2.44)	0.292
Platelet	0.44 (0.06–3.29)	0.426	–	
Glucose	4.97 (1.51–16.38)	**0.008**	2.22 (0.23–21.41)	0.489
C-reactive protein	1.09 (1.02–1.16)	**0.005**	1.02 (0.94–1.10)	0.561
Total cholesterol	0.45 (0.03–5.96)	0.552	–	
Number of sick vessels	0.60 (0.30–1.19)	0.147	–	
Rentrop (good/poor)	0.96 (0.56–1.64)	0.891	–	

OR: odds ratio; CI: confidence interval; CIN: contrast-induced nephropathy. Bold indicates significant value.

**Figure 1.**
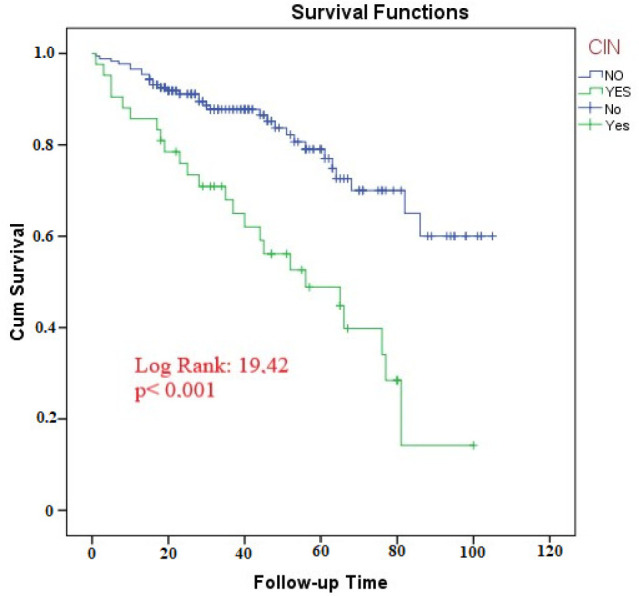
Correlation Between Development of CIN and Survival During a 100-Month Follow-Up

Kaplan-Meier analysis conducted to investigate the relationship between incidence of CIN and survival during follow-up period showed that mortality increased significantly with increasing follow-up period in patients developing CIN (log-rank p<0.001) (Figure 1).

## DISCUSSION

In this study, we identified that mortality significantly increased in long-term follow-up of patient groups requiring complex procedures such as CTO with incidence of CIN after the procedure. Development of CIN with increased procedure time in CTO lesions is an important problem. Previous studies reported its frequency in approximately 13% after coronary procedures. It has been accompanied by prolonged hospital stays, increased death rates, and costs^17^. In our study, patients who developed CIN after CTO procedures were from an older age group with lower EF rates. Though the number of patients with peripheral artery disease was relatively lower, we observed significantly more development of CIN in that group. We reported relatively more CIN incidence, albeit not significant, for patients with a history of HT, DM, chronic kidney disease (CKD), and smoking. These results are in good agreement with previous studies reported in the literature. CTO procedures are longer and more complex, requiring the use of larger volumes of contrast agent. One of the most important factors establishing the procedure time and the amount of contrast agent is the operator’s experience. Our medical centers are experienced and have been applying the procedure to CTO lesions for a long time. Hydration with normal saline before PCI continues to be the most effective approach to prevent CIN. It abates direct toxic effects of the contrast agent on epithelial cells, decreasing the concentration and viscosity of the contrast agent in tubular lumen^18^. Hydration is usually applied to patients in all risk categories; however, it is considered a requirement in the management of patients with an eGFR <60 mL/min/1.73 m^2^. Larger volume may accelerate the elimination of contrast agent, directly reduce renal toxicity, and decrease secretion of vasoconstrictors and reactive oxygen species. Many studies have demonstrated that hemoglobin, hyperglycemia, and high-sensitivity CRP are independent risk factors^19^. In our study, CRP value was significantly higher (p=0.029), and also fasting glucose values were partially higher, though not significantly, in patients with CIN. Contrast medium may enhance oxygen affinity of hemoglobin and impair oxygen transmission to peripheral tissues. Hyperglycemia may result in increased production of free oxygen radicals with increasing oxidative stress^20,21^. Further, in Mehran scoring, risk factors for mortality include DM, congestive heart failure, volume of contrast agent, age >75 years, and the use of intra-aortic balloon pump^22^. Shacham et al. conducted a study on myocardial infarction patients with preserved EF≥50 and elevated ST segment. They strongly demonstrated that older patients with poorer kidney function and history of heart failure have higher rates of mortality associated with developing acute kidney damage, hospitalization, and all causes, compared to partially younger patients with better kidney function and no heart failure^23^. Any acute reduction in kidney perfusion due to low cardiac output may result in ischemia and hypoxia in kidneys. Moreover, direct kidney damage, reactive oxygen species, and activation of sympathetic nervous system play a critical role in CIN development^24^. In our study, mortality rate in patients who developed CIN (57.1%) was higher than those who did not (42.9%). The results of our study are consistent with previous findings in the literature showing a strong association between CIN and mortality^25^. Therefore, renal functions of high-risk patients exposed to higher contrast load who have undergone a more complex and longer procedure due to conditions such as CTO must be monitored at admission and discharge. The most commonly accepted strategies in preventing CIN include exercising care in selecting patient profile and contrast volume and applying hydration.

There are limitations in this study. First, it was designed as retrospective, so bias was inevitable, and in addition, the sample size was not large enough.

## CONCLUSION

During intervention in lesions with a more complex procedure, requiring more contrast load, such as CTO, the rate of developing CIN increases in the presence of risk factors. CIN development is associated with hospital and long-term mortality. It might be possible to reduce adverse events with prophylactic approaches before the procedure and close follow-up of such patients after the procedure.
